# Crystal structure of 4-cyclo­hexyl-1-(propan-2-yl­idene)thio­semicarbazide

**DOI:** 10.1107/S160053681402025X

**Published:** 2014-09-13

**Authors:** Bohari M Yamin, Monica Lulo Rodis, Dayang N. B. A Chee

**Affiliations:** a School of Chemical Sciences and Food Technology, Faculty of Resource Science and Technology, Universiti Kebangsaan Malaysia, 43600 Bangi Selangor, Malaysia; b Department of Chemistry, Faculty of Resource Science and Technology, Universiti Malaysia Sarawak, 94300 Kota Samarahan Serawak, Malaysia

**Keywords:** crystal structure, thio­semicarbazide, thio­urea, biological activity, hydrogen bonding

## Abstract

In the title compound, C_10_H_19_N_3_S, the cyclo­hexyl group adopts a chair conformation and adopts a position approximately *syn* to the thione S atom. The CN_2_S thio­urea moiety makes dihedral angle of 13.13 (10)° with the propan-2-yl­idene­amino group. An intra­molecular N—H⋯N hydrogen bond is noted. In the crystal, inversion dimers linked by pairs of N—H⋯S hydrogen bonds generate *R* ^2^_2_(8) loops.

## Related literature   

For the applications and biological activity of thio­semicarbazide derivatives, see: Brokl *et al.* (1974 ▶), Jiang *et al.* (2006 ▶). For the crystal structures of related compounds, see: Affan *et al.* (2011 ▶); Miroslaw *et al.* (2011 ▶).
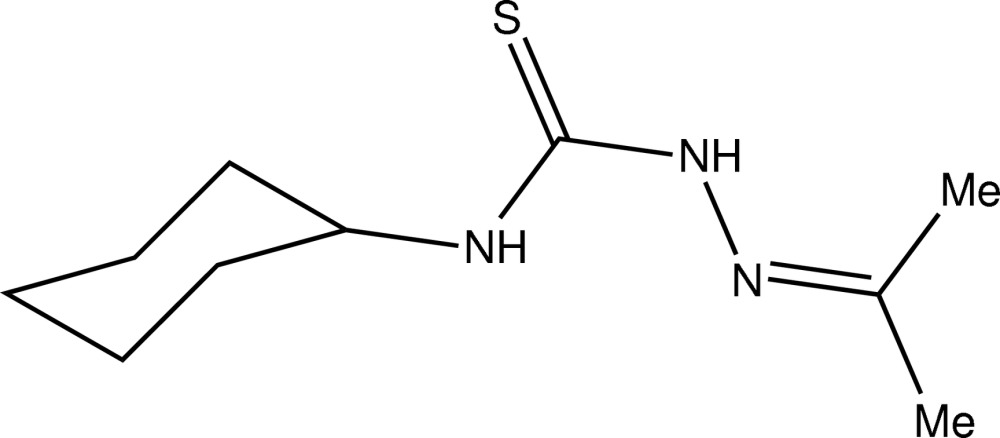


## Experimental   

### Crystal data  

C_10_H_19_N_3_S
*M* *_r_* = 213.34
Orthorhombic, 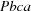

*a* = 13.6668 (10) Å
*b* = 8.3356 (5) Å
*c* = 21.4683 (16) Å
*V* = 2445.7 (3) Å^3^
*Z* = 8
Mo *K*α radiation
μ = 0.24 mm^−1^
*T* = 301 K
0.50 × 0.19 × 0.19 mm

### Data collection  

Bruker SMART APEX CCD area-detector diffractometer
Absorption correction: multi-scan (*SADABS*; Bruker, 2000 ▶) *T*_min_ = 0.892, *T*_max_ = 0.957
31768 measured reflections
3028 independent reflections
2051 reflections with *I* > 2σ(*I*)
*R*_int_ = 0.061

### Refinement  

*R*[*F* ^2^ > 2σ(*F* ^2^)] = 0.053
*wR*(*F* ^2^) = 0.135
*S* = 1.04
3028 reflections
133 parameters
H atoms treated by a mixture of independent and constrained refinement
Δρ_max_ = 0.25 e Å^−3^
Δρ_min_ = −0.30 e Å^−3^

### 

Data collection: *SMART* (Bruker, 2000 ▶); cell refinement: *SAINT* (Bruker, 2000 ▶); data reduction: *SAINT*; program(s) used to solve structure: *SHELXTL* (Sheldrick, 2008 ▶); program(s) used to refine structure: *SHELXTL*; molecular graphics: *SHELXTL*; software used to prepare material for publication: *SHELXTL* and *PLATON* (Spek, 2009 ▶).

## Supplementary Material

Crystal structure: contains datablock(s) global, I. DOI: 10.1107/S160053681402025X/tk5341sup1.cif

Structure factors: contains datablock(s) I. DOI: 10.1107/S160053681402025X/tk5341Isup2.hkl

Supporting information file. DOI: 10.1107/S160053681402025X/tk5341Isup3.cml

**Fig. 1.**
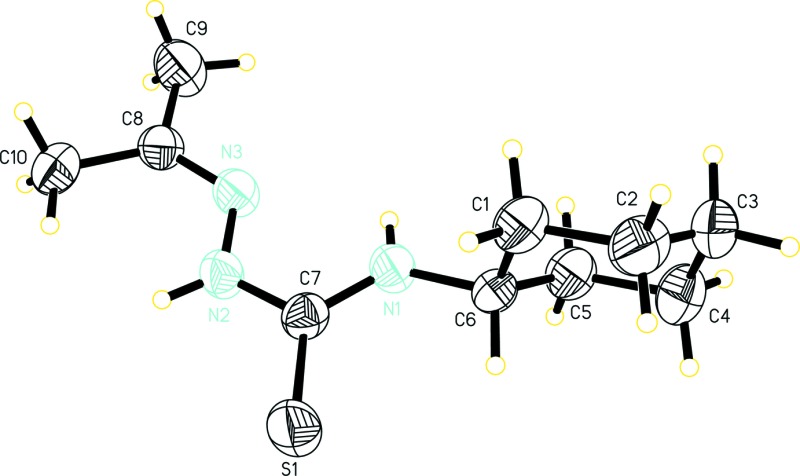
The molecular structure of (I) with displacement ellipsoids drawn at 50% probability level.

**Fig. 2.**
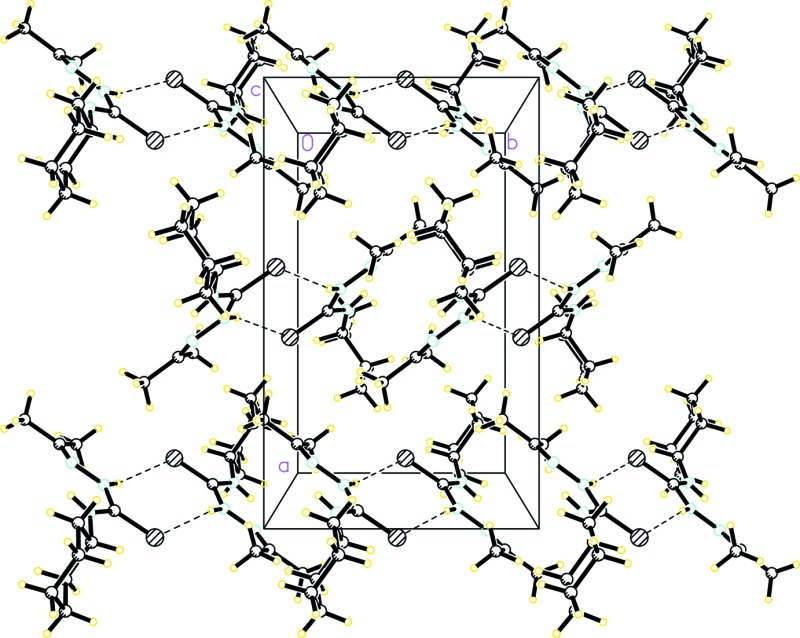
The crystal packing of (I) viewed down the c axis. The dashed lines indicate intermolecular hydrogen bonds

. DOI: 10.1107/S160053681402025X/tk5341fig1.tif
The mol­ecular structure of (I) with displacement ellipsoids drawn at 50% probability level.

c . DOI: 10.1107/S160053681402025X/tk5341fig2.tif
The crystal packing of (I) viewed down the *c* axis. The dashed lines indicate inter­molecular hydrogen bonds

CCDC reference: 1023476

Additional supporting information:  crystallographic information; 3D view; checkCIF report

## Figures and Tables

**Table 1:** Hydrogen-bond geometry (Å, °)

*D*—H⋯*A*	*D*—H	H⋯*A*	*D*⋯*A*	*D*—H⋯*A*
N1—H1*D*⋯N3	0.86	2.18	2.592 (2)	109
N2—H2*C*⋯S1^i^	0.86	2.80	3.6170 (18)	158

Symmetry code: (i) 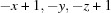.

## supplementary crystallographic information

### S5. Synthesis and crystallization

A mixture of 4-cyclo­hexyl­thio­semicarbazide (0.174 g, 1 mmol), KOH (0.112 g, 0.05 mmol) and di­phenyl­tin(IV) chloride (0.344 g, 1 mmol) in methanol was heated under reflux for 4–5 h. The reaction mixture was allowed to cool to room temperature for 1 h. The white precipitate formed was filtered and washed with acetone. Crystals suitable for X-ray study were obtained by recrystallization from acetone (0.240 g, 42% yield). *M*.pt = 390–393 K. IR (KBr): v_NH-cyclo­hexyl_ (3336), v_S=C—NH_ (3221), v_cyclo­hexyl_ (2929,2850), v_C=N_ (1527), v_C=S_ (1263,881), v_N—N_ (1106) cm^-1^. All the chemicals were purchased from Sigma Aldrich (Germany).

### S6. Refinement

Non-methine C-bound H atoms were positioned geometrically with C—H = 0.96–0.97 Å, and with *U*_iso_(H)= 1.2–1.5*U*_eq_(C). The N-bound H atoms were positioned geometrically with N—H = 0.86 Å, and with *U*_iso_(H)= 1.2*U*_eq_(N). The methine-bound H atom was located from a Fourier map and refined isotropically. A rotating model was applied in the refinement of the methyl hydrogen atoms.

### Crystal data

**Table:**

C_10_H_19_N_3_S	*F*(000) = 928
*M**_r_* = 213.34	*D*_x_ = 1.159 Mg m^−^^3^
Orthorhombic, *P**b**c**a*	Mo *K*α radiation, λ = 0.71073 Å
Hall symbol: -P 2ac 2ab	Cell parameters from 7621 reflections
*a* = 13.6668 (10) Å	θ = 2.9–28.3°
*b* = 8.3356 (5) Å	µ = 0.24 mm^−^^1^
*c* = 21.4683 (16) Å	*T* = 301 K
*V* = 2445.7 (3) Å^3^	Block, yellow
*Z* = 8	0.50 × 0.19 × 0.19 mm

### Data collection

**Table:**

Bruker SMART APEX CCD area-detector diffractometer	3028 independent reflections
Radiation source: fine-focus sealed tube	2051 reflections with *I* > 2σ(*I*)
Graphite monochromator	*R*_int_ = 0.061
Detector resolution: 83.66 pixels mm^-1^	θ_max_ = 28.3°, θ_min_ = 2.9°
ω scan	*h* = −18→16
Absorption correction: multi-scan (*SADABS*; Bruker, 2000)	*k* = −11→10
*T*_min_ = 0.892, *T*_max_ = 0.957	*l* = −28→27
31768 measured reflections	

### Refinement

**Table:**

Refinement on *F*^2^	Primary atom site location: structure-invariant direct methods
Least-squares matrix: full	Secondary atom site location: difference Fourier map
*R*[*F*^2^ > 2σ(*F*^2^)] = 0.053	Hydrogen site location: inferred from neighbouring sites
*wR*(*F*^2^) = 0.135	H atoms treated by a mixture of independent and constrained refinement
*S* = 1.04	*w* = 1/[σ^2^(*F*_o_^2^) + (0.0583*P*)^2^ + 0.9515*P*] where *P* = (*F*_o_^2^ + 2*F*_c_^2^)/3
3028 reflections	(Δ/σ)_max_ = 0.001
133 parameters	Δρ_max_ = 0.25 e Å^−^^3^
0 restraints	Δρ_min_ = −0.30 e Å^−^^3^

### Special details

**Table:**

Geometry. All e.s.d.'s (except the e.s.d. in the dihedral angle between two l.s. planes) are estimated using the full covariance matrix. The cell e.s.d.'s are taken into account individually in the estimation of e.s.d.'s in distances, angles and torsion angles; correlations between e.s.d.'s in cell parameters are only used when they are defined by crystal symmetry. An approximate (isotropic) treatment of cell e.s.d.'s is used for estimating e.s.d.'s involving l.s. planes.
Refinement. Refinement of *F*^2^ against ALL reflections. The weighted *R*-factor *wR* and goodness of fit *S* are based on *F*^2^, conventional *R*-factors *R* are based on *F*, with *F* set to zero for negative *F*^2^. The threshold expression of *F*^2^ > σ(*F*^2^) is used only for calculating *R*-factors(gt) *etc*. and is not relevant to the choice of reflections for refinement. *R*-factors based on *F*^2^ are statistically about twice as large as those based on *F*, and *R*- factors based on ALL data will be even larger.

### Fractional atomic coordinates and isotropic or equivalent isotropic displacement parameters (Å^2^)

**Table:**

	*x*	*y*	*z*	*U*_iso_*/*U*_eq_	
S1	0.59037 (4)	0.01795 (7)	0.41721 (3)	0.0582 (2)	
N1	0.51795 (12)	0.26158 (19)	0.35150 (7)	0.0477 (4)	
H1D	0.4748	0.3363	0.3483	0.057*	
N2	0.46292 (11)	0.23807 (19)	0.45107 (7)	0.0447 (4)	
H2C	0.4630	0.1942	0.4873	0.054*	
N3	0.40374 (11)	0.36924 (19)	0.43836 (7)	0.0462 (4)	
C1	0.67225 (14)	0.3396 (2)	0.30141 (9)	0.0489 (5)	
H1A	0.7093	0.3119	0.3384	0.059*	
H1B	0.6530	0.4513	0.3048	0.059*	
C2	0.73660 (16)	0.3179 (3)	0.24406 (11)	0.0580 (6)	
H2A	0.7914	0.3916	0.2463	0.070*	
H2B	0.7625	0.2096	0.2436	0.070*	
C3	0.68044 (18)	0.3477 (3)	0.18483 (10)	0.0623 (6)	
H3A	0.7223	0.3261	0.1493	0.075*	
H3B	0.6609	0.4595	0.1830	0.075*	
C4	0.59057 (17)	0.2423 (3)	0.18135 (10)	0.0589 (6)	
H4A	0.6104	0.1308	0.1786	0.071*	
H4B	0.5538	0.2682	0.1440	0.071*	
C5	0.52547 (14)	0.2654 (3)	0.23839 (9)	0.0488 (5)	
H5A	0.5001	0.3741	0.2387	0.059*	
H5B	0.4704	0.1923	0.2361	0.059*	
C6	0.58140 (13)	0.2352 (2)	0.29786 (8)	0.0393 (4)	
H1C	0.6010 (13)	0.123 (2)	0.2991 (8)	0.041 (5)*	
C7	0.52063 (13)	0.1809 (2)	0.40478 (8)	0.0387 (4)	
C8	0.36147 (14)	0.4386 (2)	0.48408 (9)	0.0426 (4)	
C9	0.29760 (19)	0.5788 (3)	0.46846 (11)	0.0714 (7)	
H9A	0.2964	0.5937	0.4241	0.107*	
H9B	0.3231	0.6736	0.4880	0.107*	
H9C	0.2324	0.5591	0.4832	0.107*	
C10	0.36946 (17)	0.3939 (3)	0.55073 (9)	0.0549 (5)	
H10A	0.4361	0.3671	0.5602	0.082*	
H10B	0.3283	0.3030	0.5590	0.082*	
H10C	0.3491	0.4825	0.5761	0.082*	

### Atomic displacement parameters (Å^2^)

**Table:**

	*U*^11^	*U*^22^	*U*^33^	*U*^12^	*U*^13^	*U*^23^
S1	0.0625 (4)	0.0585 (3)	0.0536 (3)	0.0258 (3)	0.0105 (3)	0.0124 (2)
N1	0.0531 (9)	0.0453 (9)	0.0446 (9)	0.0189 (7)	0.0155 (8)	0.0074 (7)
N2	0.0499 (9)	0.0451 (9)	0.0391 (9)	0.0104 (7)	0.0105 (7)	0.0057 (7)
N3	0.0469 (9)	0.0448 (9)	0.0468 (9)	0.0105 (7)	0.0142 (7)	0.0068 (7)
C1	0.0468 (11)	0.0532 (11)	0.0467 (11)	0.0001 (9)	−0.0012 (9)	−0.0088 (9)
C2	0.0457 (11)	0.0557 (12)	0.0725 (15)	−0.0087 (10)	0.0167 (11)	−0.0103 (11)
C3	0.0777 (15)	0.0583 (13)	0.0509 (13)	−0.0037 (11)	0.0270 (12)	−0.0024 (10)
C4	0.0682 (14)	0.0682 (14)	0.0403 (11)	0.0013 (11)	−0.0001 (10)	−0.0082 (10)
C5	0.0443 (11)	0.0535 (11)	0.0486 (12)	0.0024 (9)	0.0008 (9)	−0.0040 (9)
C6	0.0428 (10)	0.0348 (9)	0.0403 (10)	0.0084 (8)	0.0083 (8)	−0.0016 (7)
C7	0.0358 (9)	0.0391 (9)	0.0410 (10)	0.0015 (7)	0.0050 (8)	−0.0017 (8)
C8	0.0429 (10)	0.0390 (9)	0.0460 (11)	0.0000 (8)	0.0128 (8)	0.0011 (8)
C9	0.0828 (16)	0.0633 (14)	0.0682 (15)	0.0310 (13)	0.0326 (13)	0.0157 (12)
C10	0.0661 (13)	0.0528 (11)	0.0458 (12)	0.0046 (11)	0.0055 (10)	−0.0092 (9)

### Geometric parameters (Å, º)

**Table:**

S1—C7	1.6803 (18)	C3—H3B	0.9700
N1—C7	1.328 (2)	C4—C5	1.526 (3)
N1—C6	1.458 (2)	C4—H4A	0.9700
N1—H1D	0.8600	C4—H4B	0.9700
N2—C7	1.355 (2)	C5—C6	1.509 (3)
N2—N3	1.387 (2)	C5—H5A	0.9700
N2—H2C	0.8600	C5—H5B	0.9700
N3—C8	1.277 (2)	C6—H1C	0.973 (19)
C1—C6	1.518 (3)	C8—C10	1.483 (3)
C1—C2	1.524 (3)	C8—C9	1.497 (3)
C1—H1A	0.9700	C9—H9A	0.9600
C1—H1B	0.9700	C9—H9B	0.9600
C2—C3	1.506 (3)	C9—H9C	0.9600
C2—H2A	0.9700	C10—H10A	0.9600
C2—H2B	0.9700	C10—H10B	0.9600
C3—C4	1.512 (3)	C10—H10C	0.9600
C3—H3A	0.9700		
			
C7—N1—C6	125.98 (15)	C6—C5—C4	111.24 (16)
C7—N1—H1D	117.0	C6—C5—H5A	109.4
C6—N1—H1D	117.0	C4—C5—H5A	109.4
C7—N2—N3	118.20 (15)	C6—C5—H5B	109.4
C7—N2—H2C	120.9	C4—C5—H5B	109.4
N3—N2—H2C	120.9	H5A—C5—H5B	108.0
C8—N3—N2	118.00 (16)	N1—C6—C5	109.97 (14)
C6—C1—C2	111.30 (16)	N1—C6—C1	111.11 (15)
C6—C1—H1A	109.4	C5—C6—C1	111.16 (16)
C2—C1—H1A	109.4	N1—C6—H1C	106.7 (11)
C6—C1—H1B	109.4	C5—C6—H1C	108.9 (11)
C2—C1—H1B	109.4	C1—C6—H1C	108.9 (11)
H1A—C1—H1B	108.0	N1—C7—N2	115.93 (16)
C3—C2—C1	111.62 (18)	N1—C7—S1	124.19 (14)
C3—C2—H2A	109.3	N2—C7—S1	119.87 (14)
C1—C2—H2A	109.3	N3—C8—C10	126.46 (18)
C3—C2—H2B	109.3	N3—C8—C9	116.44 (18)
C1—C2—H2B	109.3	C10—C8—C9	117.10 (17)
H2A—C2—H2B	108.0	C8—C9—H9A	109.5
C2—C3—C4	111.10 (18)	C8—C9—H9B	109.5
C2—C3—H3A	109.4	H9A—C9—H9B	109.5
C4—C3—H3A	109.4	C8—C9—H9C	109.5
C2—C3—H3B	109.4	H9A—C9—H9C	109.5
C4—C3—H3B	109.4	H9B—C9—H9C	109.5
H3A—C3—H3B	108.0	C8—C10—H10A	109.5
C3—C4—C5	111.13 (17)	C8—C10—H10B	109.5
C3—C4—H4A	109.4	H10A—C10—H10B	109.5
C5—C4—H4A	109.4	C8—C10—H10C	109.5
C3—C4—H4B	109.4	H10A—C10—H10C	109.5
C5—C4—H4B	109.4	H10B—C10—H10C	109.5
H4A—C4—H4B	108.0		
			
C7—N2—N3—C8	−169.08 (17)	C2—C1—C6—N1	−177.51 (16)
C6—C1—C2—C3	54.9 (2)	C2—C1—C6—C5	−54.7 (2)
C1—C2—C3—C4	−55.4 (2)	C6—N1—C7—N2	172.31 (17)
C2—C3—C4—C5	55.8 (2)	C6—N1—C7—S1	−7.2 (3)
C3—C4—C5—C6	−55.9 (2)	N3—N2—C7—N1	2.8 (2)
C7—N1—C6—C5	145.50 (19)	N3—N2—C7—S1	−177.70 (13)
C7—N1—C6—C1	−91.0 (2)	N2—N3—C8—C10	0.4 (3)
C4—C5—C6—N1	178.77 (16)	N2—N3—C8—C9	−179.53 (18)
C4—C5—C6—C1	55.3 (2)		

### Hydrogen-bond geometry (Å, º)

**Table:**

*D*—H···*A*	*D*—H	H···*A*	*D*···*A*	*D*—H···*A*
N1—H1*D*···N3	0.86	2.18	2.592 (2)	109
C6—H1*C*···S1	0.97 (3)	2.69 (2)	3.142 (2)	108.9 (12)
C10—H10*A*···N2	0.96	2.40	2.811 (3)	106
N2—H2*C*···S1^i^	0.86	2.80	3.6170 (18)	158

Symmetry code: (i) −*x*+1, −*y*, −*z*+1.
