# Supplementary figures and images for: Crystal structure of 4-cyclo­hexyl-1-(propan-2-yl­idene)thio­semicarbazide
Source: Acta Crystallogr Sect E Struct Rep Online. 2014 Sep 13;70(Pt 10):o1109. doi: 10.1107/S160053681402025X (PMC4257229; doi:10.1107/S160053681402025X)

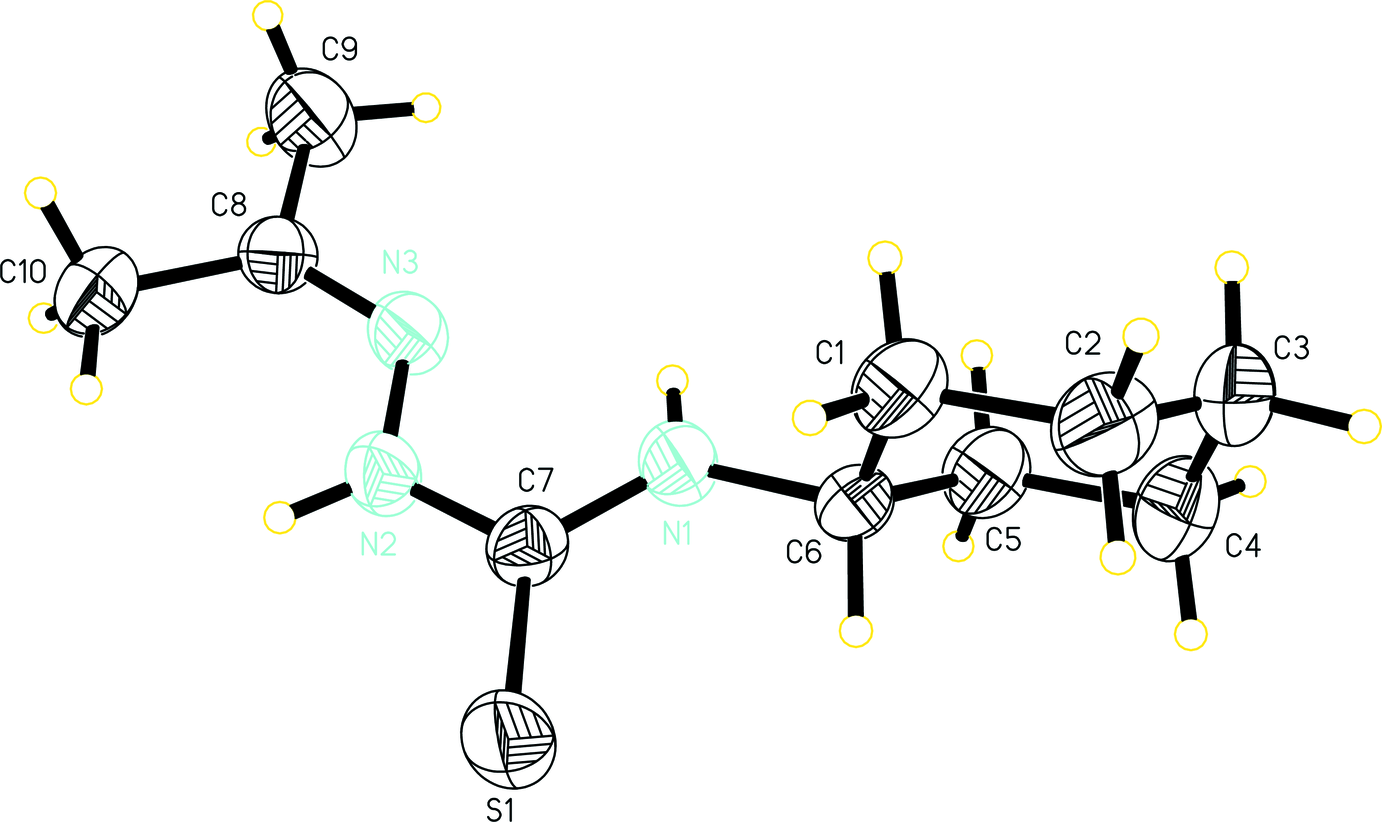

Supplement: Supplementary file 4 [file e-70-o1109-fig1.tif]

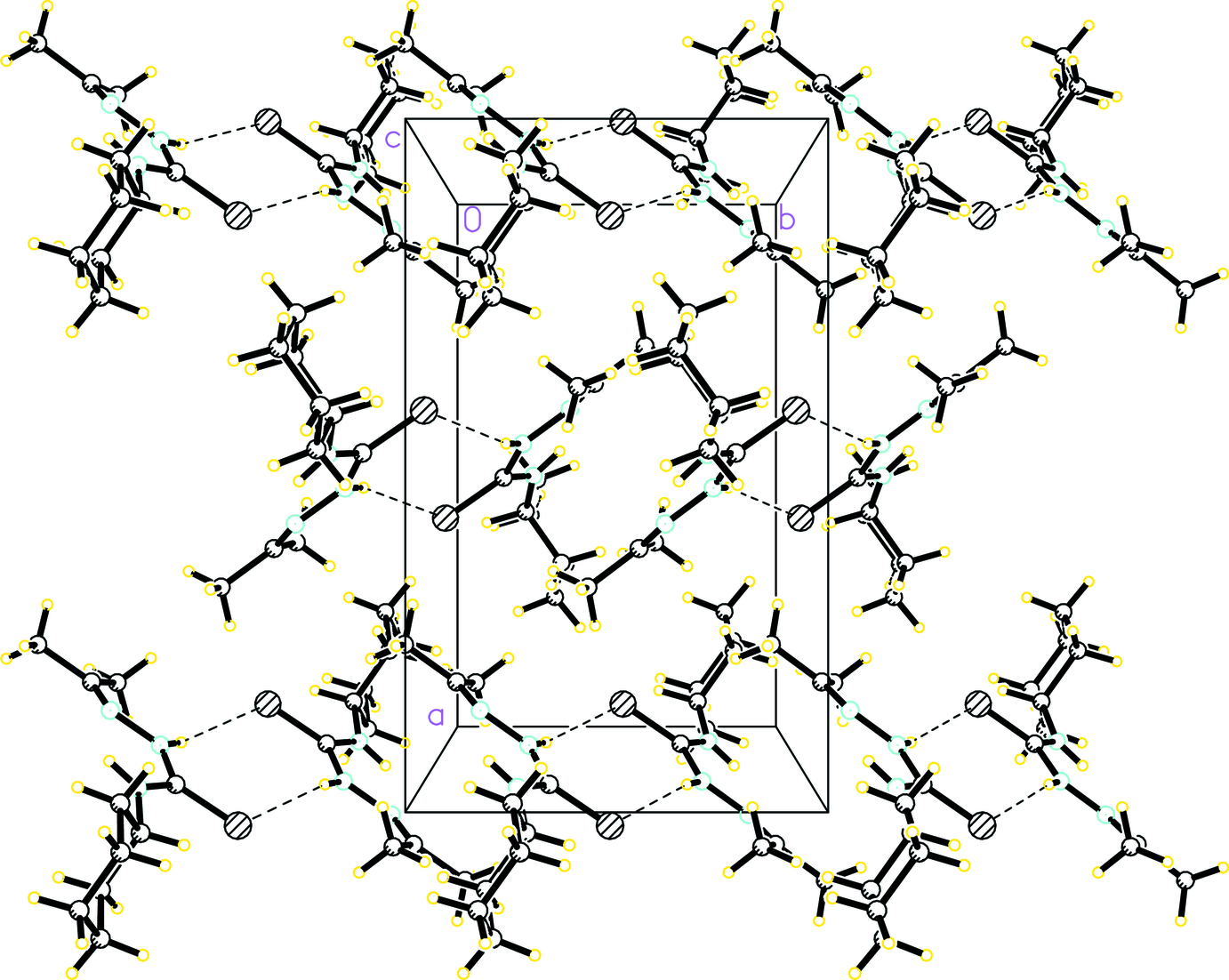

Supplement: Supplementary file 5 [file e-70-o1109-fig2.tif]
